# Accessory spleen arising from the gastric fundus mimicking gastrointestinal stromal tumor following splenectomy: A case report

**DOI:** 10.3892/etm.2013.1415

**Published:** 2013-11-19

**Authors:** GUANGYAO WANG, PING CHEN, LIANG ZONG

**Affiliations:** 1 Gastrointestinal Surgery Department, Su Bei People’s Hospital of Jiangsu Province, Yangzhou University, Yangzhou, Jiangsu 225001, P.R. China; 2 Department of Gastrointestinal Surgery, Graduate School of Medicine, University of Tokyo, Tokyo, Japan

**Keywords:** accessory spleen, gastrointestinal stromal tumor, splenectomy

## Abstract

The current case report presents an accessory spleen mimicking gastrointestinal stromal tumor (GIST) of the stomach in a patient who had undergone a splenectomy ~20 years previously. A 61-year-old male, who presented with upper abdominal discomfort lasting three months, underwent gastrointestinal endoscopy. Gastroscopy and endoscopic ultrasonography revealed a smooth, hemispherical mass of ~2 cm in diameter, with homogenous echogenicity originating from the gastric muscular layer. Abdominal contrast-enhanced computed tomography showed that the well-marginated ovoid mass was ~2.6×1.9 cm in size. The patient was diagnosed with GIST. Subsequent therapy consisted of partial gastrectomy. The pathological results indicated the mass contained splenic tissue, which confirmed it to be an accessory spleen. Changes in the postoperative platelet count were noted. The observations of this case study highlight that platelet count should be used as a routine indicator for monitoring accessory spleen arising from gastric fundus lesion.

## Introduction

The occurrence of an accessory spleen is relatively common and observed in 10–30% of autopsy patients (1,2). Accessory spleens are congenital foci of healthy splenic tissues that are separate from the main body of the spleen (3). They often originate from a failed fusion of the splenic anlage located in the dorsal mesogastrium during the 5th week of fetal development (4). Although they have been found at sites from the diaphragm to the scrotum, the vast majority are located in the spleen region, usually in the splenic hilum or along the splenic vessels or associated ligaments. The majority of accessory spleens appear as small nodules arising from adjacent organs, such as the kidney, adrenal gland and pancreas (5–10). Cases arising from the stomach are relatively rare. In the present case, the accessory spleen was unusual, presenting as a gastrointestinal stromal tumor (GIST) of the stomach at endoscopy.

This study was approved by the ethics committee of Su Bei People’s Hospital of Jiangsu Province (Yangzhou, China). The patient consented to the publication of this study.

## Case report

**Figure 1:**
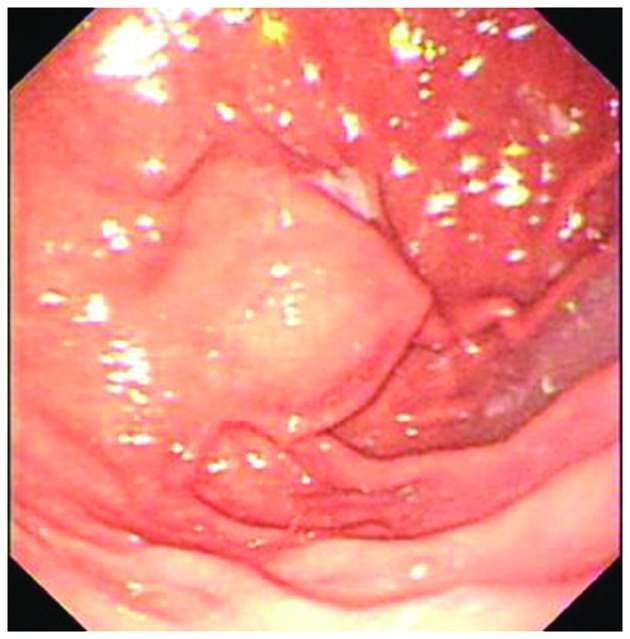
Gastrointestinal endoscopy revealed a fusiform mass at the posterior wall of the upper gastric fundus.

**Figure 2:**
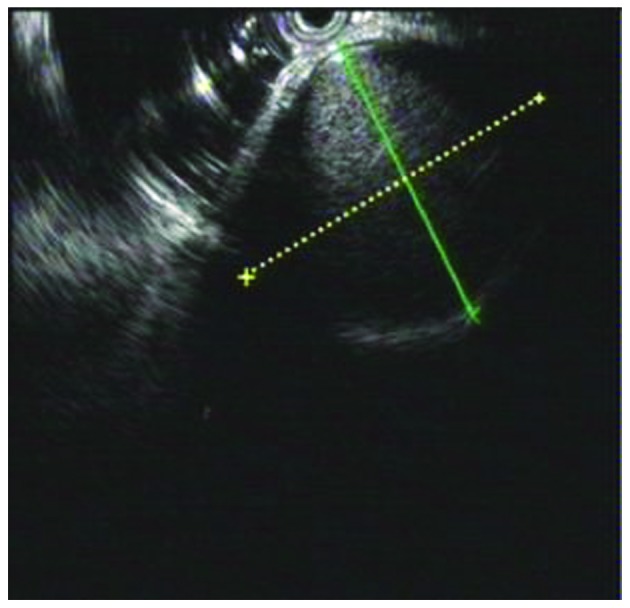
Endoscopic ultrasonography revealed a tumor with low homogenous echogenicity originating in the gastric muscular layer.

**Figure 3:**
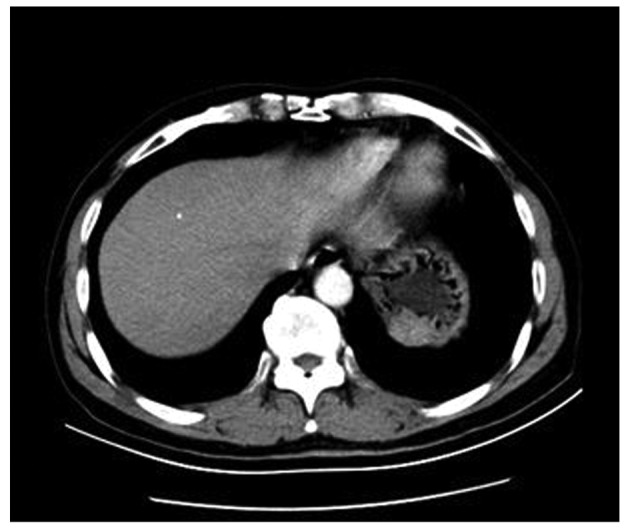
Abdominal contrast-enhanced computed tomography showed a well-marginated ovoid mass of ~2.6×1.9 cm in size located close to the gastric fundus.

**Figure 4:**
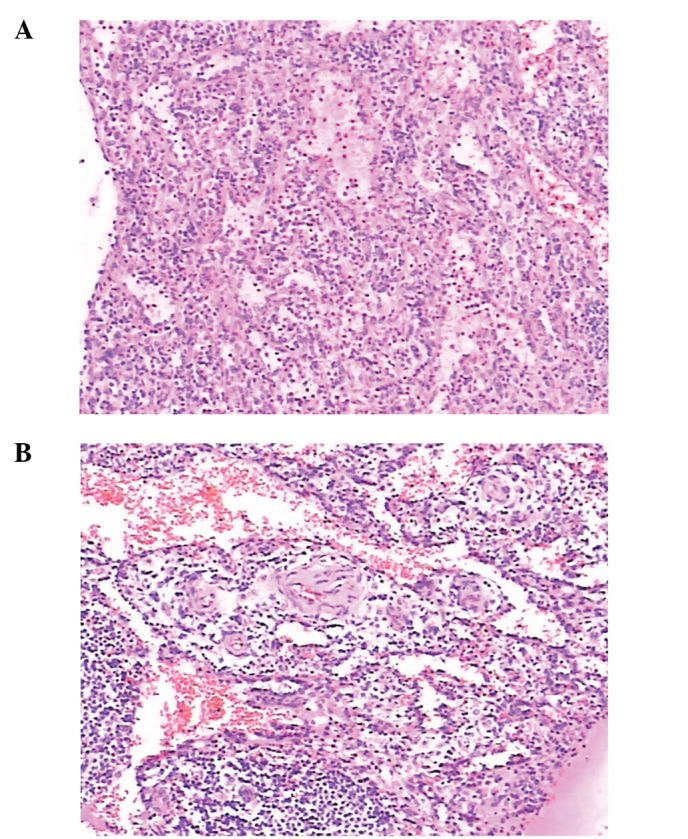
Histological examination of (A) and (B) revealed representative structures in red pulp and in the perifollicular zone of the human spleen. A number of sinusoidal spaces containing red blood cells were found to contain monocytes and lymphocytes.

A 61-year-old male was admitted to the Department of Gastroenterology of Su Bei People’s Hospital of Jiangsu Province (Yangzhou, China) presenting with an upper abdominal discomfort of 3 months in duration. Past and family histories were non-contributory and the patient did not smoke or consume alcohol and had undergone a splenectomy 20 years earlier. Upon admission, physical examination and laboratory data, including peripheral blood counts, were all unremarkable. The platelet count was 1.48×10^11^/l. The tumor markers showed no abnormalities and were as follows: Carbohydrate antigen (CA)50, 3.75 KU/l (normal range, <35.00); CA19-9, 2.09 KU/l (normal range, <35.00); α-fetoprotein, 4.58 ng/ml (<20.00); and carcinoembryonic antigen, 0.95 ng/ml (normal range, <5.00). Gastrointestinal endoscopy identified a fusiform mass at the posterior wall of the upper gastric fundus (Fig. 1). Endoscopic ultrasonography (EUS) revealed a tumor with low homogenous echogenicity originating in the gastric muscular layer (Fig. 2). Abdominal contrast-enhanced computed tomography (CT) showed a well-marginated ovoid mass ~2.6×1.9 cm in size located close to the gastric fundus (Fig. 3). A diagnosis of gastric GIST was made. Initially, endoscopic submucosal dissection was considered, however, the perforation involved rendered the problem difficult to repair by this method. Instead, gastroscopy was combined with laparoscopy. Under a laparoscope, the tumor was located at the posterior wall of the upper gastric fundus and was ~2.5×3.0 cm in size. Due to abdominal adhesions in the gastric fundus, separation and exposure was difficult. Open surgery was performed with enterolysis and partial gastrectomy. Histological examination identified specific representative structures in the red pulp and perifollicular zone of the human spleen. The tissue was largely composed of monocytes and lymphocytes and numerous sinusoidal spaces containing red blood cells were interspersed among these cells. The terminal end of the capillary branches of the arteriole sheaths were also identified, and were intermixed with lymphocytes and plasmocytes (Fig. 4). These results indicated that the mass contained splenic tissue, which confirmed it to be an accessory spleen.

## Discussion

The current case report presents the diagnosis and treatment of an accessory spleen adjoining the stomach fundus, which appeared as a GIST at endoscopy in a patient who had undergone a splenectomy 20 years earlier. It has been reported that accessory spleens can have compensatory hypertrophy of residual splenic tissue following splenectomy and occasionally reach 3–5 cm in size (2). The accessory spleen observed in the present case appeared to be a GIST, considering that the patient had a history of splenectomy.

The following diagnostic approach can be considered effective when the diagnosis is unclear. CT is an important imaging technique used to evaluate the abdomen. It can identify the shape of accessory spleens (oval or round) and whether attenuation is identical to that of a proper splenic parenchyma prior to and following administration of contrast medium (1). Familiarity with the CT features of accessory spleens is useful to determine a diagnosis (6). Typically, accessory spleens are round or oval and the attenuation is identical to that of the proper splenic parenchyma prior to and following administration of contrast medium (6). Vascular branches arising from the splenic artery can be observed on dynamic CT (1). Endoscopic ultrasonography is able to show whether a mass with the homogenous parenchymal texture has originated from extragastric tissue, such as splenic parenchyma. The patient in the present case study had previously undergone a splenectomy, therefore it was impossible to compare the accessory spleen to proper splenic parenchyma. EUS-guided fine needle aspiration is beneficial for diagnosis of accessory spleen, which mimics a gastric subepithelial mass observed in histological examination (11). However, certain accessory spleens mimic an enlarged lymph node or tumor arising from adjacent organs, such as the kidney, adrenal gland or pancreas (12–15). Similarly, accessory spleens may be differentiated from metastatic lesions or lymphadenopathy when they are enhanced to the same degree as the spleen (1). In such cases, technetium 99m sulfur colloid scintigraphy provides an easy method of establishing the identity of ectopic splenic tissues (5,6). The mass mimicking GIST was readily identified by radionuclide imaging in the present case, resulting in the definite diagnosis of accessory spleens. An accessory spleen should be suspected in this type of case. In addition, ectopic splenic tissue may be caused by autotransplantation of splenic cells within the peritoneal cavity resulting from traumatic disruption of the splenic capsule (16,17).

Although an accessory spleen is usually found incidentally with no clinical significance in the majority of patients (1,6), it may occasionally be relevant to detection and characterization in clinical situations (18–20). Accessory spleens may become symptomatic due to spontaneous rupture, hemorrhage, embolism or torsion. The clinical significance of a residual accessory spleen post-splenectomy varies according to the individual conditions. Surgeons must be aware of their presence when the intention is to remove functional splenic tissues. The return of splenic function caused by compensatory enlargement of ectopic splenic tissues has been implicated in the recurrence of hematological disorders, such as thrombocytopenic purpura (18–20).

During follow-up, the platelet count of the present patient increased to 3.92×10^11^/l on the second day following surgery and a high level of 4.10×10^11^/l was observed on day 9. The patient peripheral blood count returned to normal after 2 weeks, and there was no evidence of recurrence. Platelet count usually increases within 2–3 days of splenectomy, peaks between 7 and 14 days and then gradually decreases and returns to normal after 1–2 months (21). This condition may cause venous thrombosis if the platelet count increases abnormally. Once thrombosis has extended to the superior mesenteric vein, it may cause extensive necrosis of its convolutions. Similarly, high postoperative platelet counts can easily lead to deep venous thrombosis of the lower limbs, resulting in pulmonary embolism and even mortality. For this reason, changes in platelet count should be observed carefully following a splenectomy. Appropriate treatment based on the platelet count and liver function may provide good therapeutic effects.

In conclusion, patients who present with accessory spleen arising from gastric fundus following splenectomy should undergo careful follow-up by imaging examination, including CT. If the clinical course is uneventful and the patient remains asymptomatic without any abnormalities in physical and laboratory examinations, then splenectomy should not be performed. Changes in postoperative platelet count should be taken into account. If splenectomy is necessary, platelet count should be used as a routine monitoring indicator.
